# 8-Methyl-4-phenyl-2,3,3a,4,5,9b-hexa­hydro­furo[3,2-*c*]quinoline

**DOI:** 10.1107/S1600536809051125

**Published:** 2009-12-04

**Authors:** Pingping Lu, Chaomei Lian, Yulin Zhu

**Affiliations:** a School of Chemistry and Environment, South China Normal University, Guangzhou 510006, People’s Republic of China

## Abstract

The title compound, C_18_H_19_NO, was synthesized from the multi-component one-pot reaction between *p*-toluidine, benzaldehyde and 2,3-dihydro­furan in the presence of palladium dichloride. There are two mol­ecules in the asymmetric unit. The crystal packing is stabilized by classical inter­molecular N—H⋯O hydrogen bonds.

## Related literature

For heterocyclic scaffolds of biologically active alkaloids, see: Johnson *et al.* (1989 ▶); Yamada *et al.* (1992 ▶); Katritzky & Rachwal (1996 ▶). For the synthesis of related compounds, see: Buonora *et al.* (2001 ▶); Syamala (2005 ▶).
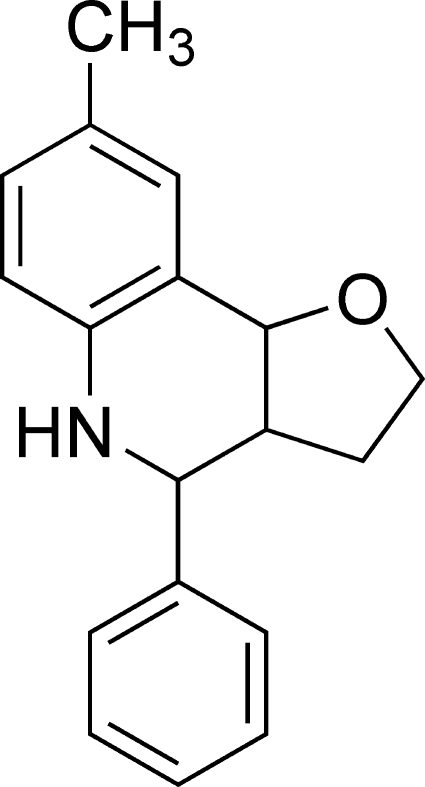


## Experimental

### 

#### Crystal data

C_18_H_19_NO
*M* *_r_* = 265.34
Monoclinic, 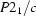

*a* = 12.751 (4) Å
*b* = 17.780 (5) Å
*c* = 17.516 (4) Å
β = 132.426 (14)°
*V* = 2931.3 (15) Å^3^
*Z* = 8
Mo *K*α radiation
μ = 0.07 mm^−1^
*T* = 295 K
0.30 × 0.15 × 0.15 mm

#### Data collection

Bruker APEXII area-detector diffractometer
Absorption correction: multi-scan (*SADABS*; Bruker, 2004 ▶) *T*_min_ = 0.987, *T*_max_ = 0.989
14911 measured reflections
5280 independent reflections
2114 reflections with *I* > 2σ(*I*)
*R*_int_ = 0.067

#### Refinement

*R*[*F* ^2^ > 2σ(*F* ^2^)] = 0.069
*wR*(*F* ^2^) = 0.186
*S* = 1.17
5280 reflections
315 parameters
H-atom parameters constrained
Δρ_max_ = 0.22 e Å^−3^
Δρ_min_ = −0.21 e Å^−3^

### 

Data collection: *APEX2* (Bruker, 2004 ▶); cell refinement: *SAINT* (Bruker, 2004 ▶); data reduction: *SAINT*; program(s) used to solve structure: *SHELXS97* (Sheldrick, 2008 ▶); program(s) used to refine structure: *SHELXL97* (Sheldrick, 2008 ▶); molecular graphics: *SHELXTL* (Sheldrick, 2008 ▶); software used to prepare material for publication: *SHELXTL*.

## Supplementary Material

Crystal structure: contains datablocks global, I. DOI: 10.1107/S1600536809051125/rk2180sup1.cif

Structure factors: contains datablocks I. DOI: 10.1107/S1600536809051125/rk2180Isup2.hkl

Additional supplementary materials:  crystallographic information; 3D view; checkCIF report

## Figures and Tables

**Table 1:** Hydrogen-bond geometry (Å, °)

*D*—H⋯*A*	*D*—H	H⋯*A*	*D*⋯*A*	*D*—H⋯*A*
N1—H1⋯O2	0.86	2.41	2.959 (4)	122
N2—H2⋯O1^i^	0.86	2.15	2.934 (4)	151

Symmetry code: (i) 
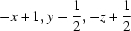
.

**Fig. 1.**
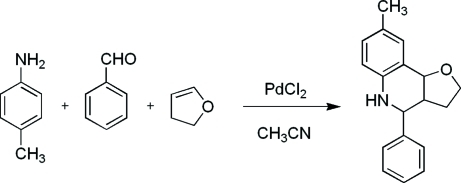
Palladium dichloride catalyzed synthesis of the title compound.

**Fig. 2.**
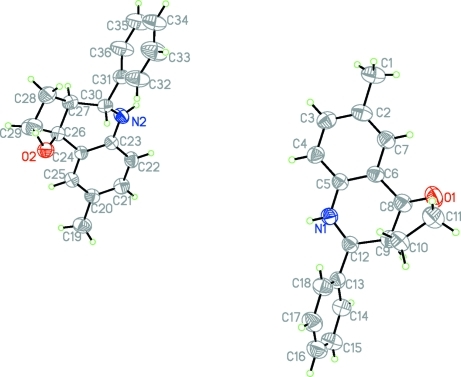
View of the title compound showing the atom-labelling scheme. Displacement ellipsoids are drawn at the 50% probability level. H atoms are presented as a small spheres of arbitrary radius.

## supplementary crystallographic information

### Comment

Tetrahydroquinolines are well known as important heterocyclic scaffolds in many biologically active alkaloids, examples including *flindersine*, *oricine* and *verprisine* (Johnson *et al.*, 1989; Katritzky & Rachwal, 1996; Yamada *et al.*, 1992). Aza Diels-Alder reaction which a one-pot condensation of aryl amine, aromatic aldehydes and 2,3-dihydrofuran or 3,4-dihydro-2*H*-pyran is a wellestablished method used for the construction of tetrahydroquinolines (Buonora *et al.*, 2001; Syamala, 2005). The reaction between *p*-toluidine, benzaldehyde and 2,3-dihydrofuran in the presence of palladium dichloride proceeded to give the title compound in isolated yield 92.6% (Fig. 1). A representation of the title compound is given in Fig. 2. There are no unusual bond lengths and angles in the compound and the *trans*- and *cis*-conformations were both formed in the reaction. The compound contains two different size rings: the tetrahydropyranoquinoline ring connected a phenyl ring, the structure about two rings connected each other *via* C12–C13 and C30–C31 bonds. In addition, the molecules in the structure are linked *via* intermolecular hydrogen bonds N1–H1···O2 and N2–H2···O1^i^. Symmetry code: (i) -*x*+1, *y*-1/2, -*z*+1/2.

### Experimental

A mixture of *p*-toluidine (1.07 g, 10 mmol), benzaldehyde (1.06 g, 10 mmol), 2,3-dihydrofuran (0.84 g, 12 mmol), and palladium dichloride (0.0020 mg) was refluxed in acetonitrile (12 ml) at 373 K for 10 h. After being cooled to room temperature, the reaction mixture was poured into water. The white precipitate was filtered off with a silica pad, washed twice with water, and the filtrate was then dried under vacuum. Yield 92.6%. Single crystals of the title compound were obtained by slow evaporation from ethanol at room temperature to yield colourless, block-shaped crystal.

### Refinement

The H atoms were positioned geometrically and allowed to ride on their parent atoms, with C–H = 0.93-0.98Å and N–H = 0.86Å, respectively, and *U*_iso_ = 1.2 or 1.5U_eq_(parent atom).

### Crystal data

**Table:**

C_18_H_19_NO	*F*(000) = 1136
*M**_r_* = 265.34	*D*_x_ = 1.202 Mg m^−^^3^
Monoclinic, *P*2_1_/*c*	Mo *K*α radiation, λ = 0.71073 Å
Hall symbol: -P 2ybc	Cell parameters from 1233 reflections
*a* = 12.751 (4) Å	θ = 2.3–18.3°
*b* = 17.780 (5) Å	µ = 0.07 mm^−^^1^
*c* = 17.516 (4) Å	*T* = 295 K
β = 132.426 (14)°	Block, colourless
*V* = 2931.3 (15) Å^3^	0.30 × 0.15 × 0.15 mm
*Z* = 8	

### Data collection

**Table:**

Bruker APEXII area-detector diffractometer	5280 independent reflections
Radiation source: fine-focus sealed tube	2114 reflections with *I* > 2σ(*I*)
graphite	*R*_int_ = 0.067
φ and ω scans	θ_max_ = 25.2°, θ_min_ = 2.0°
Absorption correction: multi-scan (*SADABS*; Bruker, 2004)	*h* = −15→13
*T*_min_ = 0.987, *T*_max_ = 0.989	*k* = −19→21
14911 measured reflections	*l* = −19→20

### Refinement

**Table:**

Refinement on *F*^2^	Primary atom site location: structure-invariant direct methods
Least-squares matrix: full	Secondary atom site location: difference Fourier map
*R*[*F*^2^ > 2σ(*F*^2^)] = 0.069	Hydrogen site location: inferred from neighbouring sites
*wR*(*F*^2^) = 0.186	H-atom parameters constrained
*S* = 1.17	*w* = 1/[σ^2^(*F*_o_^2^) + (0.045*P*)^2^ + 0.45*P*] where *P* = (*F*_o_^2^ + 2*F*_c_^2^)/3
5280 reflections	(Δ/σ)_max_ < 0.001
315 parameters	Δρ_max_ = 0.22 e Å^−^^3^
0 restraints	Δρ_min_ = −0.21 e Å^−^^3^

### Special details

**Table:**

Geometry. All s.u.'s (except the esd in the dihedral angle between two l.s. planes) are estimated using the full covariance matrix. The cell s.u.'s are taken into account individually in the estimation of s.u.'s in distances, angles and torsion angles; correlations between s.u.'s in cell parameters are only used when they are defined by crystal symmetry. An approximate (isotropic) treatment of cell s.u.'s is used for estimating s.u.'s involving l.s. planes.
Refinement. Refinement of *F*^2^ against ALL reflections. The weighted *R*-factor w*R* and goodness of fit *S* are based on *F*^2^, conventional *R*-factors *R* are based on *F*, with *F* set to zero for negative *F*^2^. The threshold expression of *F*^2^ > 2σ(*F*^2^) is used only for calculating *R*-factors(gt) etc. and is not relevant to the choice of reflections for refinement. *R*-factors based on *F*^2^ are statistically about twice as large as those based on *F*, and *R*-factors based on ALL data will be even larger.

### Fractional atomic coordinates and isotropic or equivalent isotropic displacement parameters (Å^2^)

**Table:**

	*x*	*y*	*z*	*U*_iso_*/*U*_eq_	
N1	0.6400 (3)	0.92041 (17)	0.1751 (2)	0.0704 (9)	
H1	0.6805	0.8939	0.1601	0.084*	
C1	0.8237 (5)	1.2230 (2)	0.3116 (3)	0.1063 (16)	
H1A	0.8247	1.2483	0.2637	0.159*	
H1B	0.9177	1.2230	0.3790	0.159*	
H1C	0.7600	1.2485	0.3144	0.159*	
C2	0.7734 (4)	1.14134 (12)	0.2756 (2)	0.0783 (12)	
C3	0.8651 (2)	1.08924 (18)	0.28789 (19)	0.0806 (12)	
H3	0.9558	1.1038	0.3175	0.097*	
C4	0.8212 (3)	1.01531 (16)	0.2559 (2)	0.0746 (11)	
H4	0.8825	0.9804	0.2641	0.090*	
C5	0.6856 (3)	0.99347 (11)	0.2116 (2)	0.0636 (10)	
C6	0.5939 (2)	1.04557 (16)	0.19939 (19)	0.0698 (11)	
C7	0.6378 (3)	1.11950 (14)	0.2314 (2)	0.0787 (12)	
H7	0.5765	1.1544	0.2232	0.094*	
O1	0.3443 (3)	1.07237 (16)	0.0794 (3)	0.1021 (10)	
C8	0.4479 (5)	1.0230 (2)	0.1582 (3)	0.0808 (12)	
H8	0.4482	1.0236	0.2143	0.097*	
C9	0.4029 (4)	0.9456 (2)	0.1070 (3)	0.0746 (11)	
H9	0.3305	0.9255	0.1061	0.090*	
C10	0.3334 (5)	0.9635 (2)	−0.0018 (3)	0.0834 (12)	
H10A	0.2570	0.9284	−0.0501	0.100*	
H10B	0.4017	0.9616	−0.0097	0.100*	
C11	0.2772 (5)	1.0417 (3)	−0.0188 (4)	0.1070 (15)	
H11A	0.2980	1.0721	−0.0532	0.128*	
H11B	0.1750	1.0405	−0.0618	0.128*	
C12	0.5249 (4)	0.8892 (2)	0.1623 (3)	0.0691 (11)	
H12	0.5598	0.8810	0.2315	0.083*	
C13	0.4766 (3)	0.81442 (12)	0.10806 (19)	0.0670 (10)	
C14	0.4233 (3)	0.76150 (17)	0.13274 (19)	0.0819 (12)	
H14	0.4225	0.7717	0.1844	0.098*	
C15	0.3711 (3)	0.69334 (14)	0.0802 (2)	0.0932 (14)	
H15	0.3354	0.6579	0.0967	0.112*	
C16	0.3723 (3)	0.67811 (13)	0.0029 (2)	0.0945 (14)	
H16	0.3374	0.6325	−0.0322	0.113*	
C17	0.4257 (3)	0.73103 (17)	−0.02174 (18)	0.0928 (14)	
H17	0.4265	0.7208	−0.0734	0.111*	
C18	0.4778 (3)	0.79919 (15)	0.0308 (2)	0.0805 (12)	
H18	0.5135	0.8346	0.0143	0.097*	
O2	0.8981 (3)	0.82604 (14)	0.2907 (2)	0.0841 (8)	
N2	0.7998 (3)	0.68166 (16)	0.3925 (2)	0.0739 (9)	
H2	0.7892	0.6442	0.4179	0.089*	
C19	0.7740 (5)	0.9864 (2)	0.4799 (4)	0.1011 (15)	
H19A	0.7976	0.9903	0.5447	0.152*	
H19B	0.6787	1.0040	0.4252	0.152*	
H19C	0.8387	1.0164	0.4823	0.152*	
C20	0.7851 (3)	0.90401 (11)	0.46008 (18)	0.0713 (11)	
C21	0.7154 (3)	0.84833 (16)	0.46715 (19)	0.0805 (12)	
H21	0.6648	0.8609	0.4861	0.097*	
C22	0.7215 (3)	0.77394 (14)	0.4459 (2)	0.0766 (12)	
H22	0.6749	0.7367	0.4507	0.092*	
C23	0.7971 (3)	0.75522 (10)	0.41766 (18)	0.0646 (10)	
C24	0.8668 (2)	0.81089 (14)	0.41060 (18)	0.0607 (10)	
C25	0.8607 (2)	0.88529 (12)	0.43181 (19)	0.0695 (11)	
H25	0.9073	0.9225	0.4271	0.083*	
C26	0.9540 (4)	0.7911 (2)	0.3858 (3)	0.0722 (11)	
H26	1.0510	0.8094	0.4417	0.087*	
C27	0.9609 (4)	0.7077 (2)	0.3691 (3)	0.0779 (12)	
H27	1.0399	0.6837	0.4350	0.093*	
C28	0.9901 (5)	0.7086 (2)	0.2974 (4)	0.1086 (16)	
H28A	1.0894	0.6983	0.3359	0.130*	
H28B	0.9324	0.6713	0.2431	0.130*	
C29	0.9512 (6)	0.7860 (3)	0.2531 (4)	0.1208 (19)	
H29A	1.0341	0.8114	0.2733	0.145*	
H29B	0.8796	0.7836	0.1782	0.145*	
C30	0.8210 (4)	0.67054 (19)	0.3224 (3)	0.0657 (10)	
H30	0.7451	0.6977	0.2586	0.079*	
C31	0.8059 (4)	0.58845 (13)	0.2948 (3)	0.0754 (12)	
C32	0.6928 (3)	0.56536 (18)	0.1940 (2)	0.1154 (18)	
H32	0.6292	0.6007	0.1436	0.138*	
C33	0.6748 (4)	0.4895 (2)	0.1684 (3)	0.140 (2)	
H33	0.5992	0.4741	0.1009	0.168*	
C34	0.7699 (5)	0.43679 (13)	0.2437 (4)	0.1257 (19)	
H34	0.7579	0.3861	0.2266	0.151*	
C35	0.8830 (4)	0.45988 (17)	0.3446 (3)	0.133 (2)	
H35	0.9466	0.4246	0.3950	0.159*	
C36	0.9010 (3)	0.5357 (2)	0.3702 (2)	0.1198 (18)	
H36	0.9766	0.5511	0.4377	0.144*	

### Atomic displacement parameters (Å^2^)

**Table:**

	*U*^11^	*U*^22^	*U*^33^	*U*^12^	*U*^13^	*U*^23^
C23	0.066 (3)	0.060 (3)	0.070 (3)	0.000 (2)	0.047 (2)	0.002 (2)
C24	0.058 (3)	0.059 (2)	0.063 (2)	−0.0010 (19)	0.040 (2)	0.0028 (19)
C25	0.073 (3)	0.066 (3)	0.071 (3)	−0.006 (2)	0.049 (2)	−0.002 (2)
C20	0.075 (3)	0.065 (3)	0.065 (3)	−0.004 (2)	0.044 (2)	−0.010 (2)
C21	0.092 (3)	0.085 (3)	0.079 (3)	−0.002 (2)	0.063 (3)	−0.010 (2)
C22	0.085 (3)	0.078 (3)	0.088 (3)	−0.010 (2)	0.066 (3)	−0.006 (2)
C5	0.066 (3)	0.063 (3)	0.054 (2)	0.005 (2)	0.037 (2)	0.004 (2)
C6	0.075 (3)	0.064 (3)	0.065 (3)	−0.004 (2)	0.045 (3)	−0.004 (2)
C7	0.087 (3)	0.073 (3)	0.076 (3)	0.000 (2)	0.055 (3)	−0.007 (2)
C2	0.107 (4)	0.066 (3)	0.064 (3)	−0.010 (3)	0.058 (3)	0.003 (2)
C3	0.081 (3)	0.086 (3)	0.072 (3)	−0.007 (3)	0.051 (3)	0.011 (2)
C4	0.071 (3)	0.077 (3)	0.078 (3)	0.000 (2)	0.051 (3)	0.009 (2)
C13	0.059 (3)	0.072 (3)	0.058 (3)	0.004 (2)	0.035 (2)	−0.006 (2)
C14	0.088 (3)	0.074 (3)	0.084 (3)	−0.004 (2)	0.058 (3)	−0.003 (2)
C15	0.112 (4)	0.070 (3)	0.093 (3)	−0.009 (3)	0.067 (3)	−0.004 (3)
C16	0.099 (4)	0.074 (3)	0.088 (4)	−0.004 (2)	0.054 (3)	−0.014 (3)
C17	0.106 (4)	0.086 (3)	0.080 (3)	0.001 (3)	0.060 (3)	−0.015 (3)
C18	0.097 (3)	0.076 (3)	0.077 (3)	−0.007 (2)	0.062 (3)	−0.010 (2)
C31	0.086 (3)	0.065 (3)	0.086 (3)	0.011 (2)	0.062 (3)	0.004 (2)
C36	0.127 (4)	0.068 (3)	0.105 (4)	0.022 (3)	0.054 (4)	0.006 (3)
C35	0.156 (5)	0.082 (4)	0.130 (5)	0.027 (3)	0.084 (5)	0.011 (3)
C34	0.161 (6)	0.071 (3)	0.148 (5)	0.016 (4)	0.105 (5)	−0.012 (4)
C33	0.163 (6)	0.091 (4)	0.111 (5)	0.001 (4)	0.070 (4)	−0.029 (4)
C32	0.131 (5)	0.085 (4)	0.090 (4)	0.021 (3)	0.058 (4)	−0.012 (3)
O1	0.100 (2)	0.084 (2)	0.111 (3)	0.0195 (18)	0.067 (2)	−0.007 (2)
N1	0.078 (2)	0.068 (2)	0.080 (2)	−0.0036 (17)	0.060 (2)	−0.0116 (17)
C1	0.131 (4)	0.080 (3)	0.096 (4)	−0.023 (3)	0.072 (3)	−0.002 (3)
C8	0.090 (4)	0.074 (3)	0.076 (3)	0.002 (3)	0.055 (3)	−0.001 (3)
C9	0.081 (3)	0.076 (3)	0.079 (3)	−0.003 (2)	0.059 (3)	−0.003 (2)
C10	0.105 (3)	0.068 (3)	0.082 (3)	0.010 (2)	0.065 (3)	0.001 (2)
C11	0.119 (4)	0.093 (4)	0.100 (4)	0.006 (3)	0.070 (4)	0.002 (3)
C12	0.072 (3)	0.066 (3)	0.069 (3)	0.001 (2)	0.047 (2)	−0.002 (2)
O2	0.101 (2)	0.0803 (19)	0.106 (2)	0.0212 (15)	0.084 (2)	0.0236 (17)
N2	0.099 (3)	0.058 (2)	0.089 (2)	−0.0049 (17)	0.074 (2)	−0.0011 (18)
C19	0.124 (4)	0.081 (3)	0.111 (4)	0.007 (3)	0.085 (3)	−0.010 (3)
C26	0.069 (3)	0.067 (3)	0.086 (3)	0.002 (2)	0.054 (3)	0.005 (2)
C27	0.073 (3)	0.075 (3)	0.088 (3)	0.012 (2)	0.056 (3)	0.012 (2)
C28	0.135 (4)	0.091 (4)	0.162 (5)	0.015 (3)	0.126 (4)	0.006 (3)
C29	0.169 (5)	0.113 (4)	0.159 (5)	0.049 (4)	0.142 (5)	0.037 (4)
C30	0.069 (3)	0.061 (2)	0.070 (3)	0.007 (2)	0.048 (2)	0.000 (2)

### Geometric parameters (Å, °)

**Table:**

C23—N2	1.388 (3)	C35—H35	0.9300
C23—C24	1.3900	C34—C33	1.3900
C23—C22	1.3900	C34—H34	0.9300
C24—C25	1.3900	C33—C32	1.3900
C24—C26	1.484 (4)	C33—H33	0.9300
C25—C20	1.3900	C32—H32	0.9300
C25—H25	0.9300	O1—C8	1.400 (4)
C20—C21	1.3900	O1—C11	1.415 (5)
C20—C19	1.534 (4)	N1—C12	1.438 (4)
C21—C22	1.3900	N1—H1	0.8600
C21—H21	0.9300	C1—H1A	0.9600
C22—H22	0.9300	C1—H1B	0.9600
C5—C6	1.3900	C1—H1C	0.9600
C5—C4	1.3900	C8—C9	1.526 (5)
C5—N1	1.391 (3)	C8—H8	0.9800
C6—C7	1.3900	C9—C10	1.497 (5)
C6—C8	1.528 (5)	C9—C12	1.527 (5)
C7—C2	1.3900	C9—H9	0.9800
C7—H7	0.9300	C10—C11	1.500 (5)
C2—C3	1.3900	C10—H10A	0.9700
C2—C1	1.539 (4)	C10—H10B	0.9700
C3—C4	1.3900	C11—H11A	0.9700
C3—H3	0.9300	C11—H11B	0.9700
C4—H4	0.9300	C12—H12	0.9800
C13—C14	1.3900	O2—C29	1.415 (9)
C13—C18	1.3900	O2—C26	1.438 (4)
C13—C12	1.504 (4)	N2—C30	1.438 (4)
C14—C15	1.3900	N2—H2	0.8599
C14—H14	0.9300	C19—H19A	0.9600
C15—C16	1.3900	C19—H19B	0.9600
C15—H15	0.9300	C19—H19C	0.9600
C16—C17	1.3900	C26—C27	1.525 (5)
C16—H16	0.9300	C26—H26	0.9800
C17—C18	1.3900	C27—C30	1.522 (5)
C17—H17	0.9300	C27—C28	1.532 (5)
C18—H18	0.9300	C27—H27	0.9800
C31—C36	1.3900	C28—C29	1.491 (5)
C31—C32	1.3900	C28—H28A	0.9700
C31—C30	1.508 (4)	C28—H28B	0.9700
C36—C35	1.3900	C29—H29A	0.9700
C36—H36	0.9300	C29—H29B	0.9700
C35—C34	1.3900	C30—H30	0.9800
			
N2—C23—C24	119.3 (2)	C2—C1—H1A	109.5
N2—C23—C22	120.7 (2)	C2—C1—H1B	109.5
C24—C23—C22	120.0	H1A—C1—H1B	109.5
C25—C24—C23	120.0	C2—C1—H1C	109.5
C25—C24—C26	119.5 (2)	H1A—C1—H1C	109.5
C23—C24—C26	120.5 (2)	H1B—C1—H1C	109.5
C24—C25—C20	120.0	O1—C8—C9	104.8 (3)
C24—C25—H25	120.0	O1—C8—C6	109.8 (3)
C20—C25—H25	120.0	C9—C8—C6	111.7 (3)
C21—C20—C25	120.0	O1—C8—H8	110.1
C21—C20—C19	119.9 (2)	C9—C8—H8	110.1
C25—C20—C19	120.1 (2)	C6—C8—H8	110.1
C20—C21—C22	120.0	C10—C9—C8	102.8 (3)
C20—C21—H21	120.0	C10—C9—C12	114.9 (3)
C22—C21—H21	120.0	C8—C9—C12	113.6 (3)
C21—C22—C23	120.0	C10—C9—H9	108.4
C21—C22—H22	120.0	C8—C9—H9	108.4
C23—C22—H22	120.0	C12—C9—H9	108.4
C6—C5—C4	120.0	C9—C10—C11	104.0 (3)
C6—C5—N1	120.3 (2)	C9—C10—H10A	111.0
C4—C5—N1	119.6 (2)	C11—C10—H10A	111.0
C5—C6—C7	120.0	C9—C10—H10B	111.0
C5—C6—C8	121.8 (2)	C11—C10—H10B	111.0
C7—C6—C8	118.0 (2)	H10A—C10—H10B	109.0
C2—C7—C6	120.0	O1—C11—C10	107.5 (4)
C2—C7—H7	120.0	O1—C11—H11A	110.2
C6—C7—H7	120.0	C10—C11—H11A	110.2
C7—C2—C3	120.0	O1—C11—H11B	110.2
C7—C2—C1	120.6 (3)	C10—C11—H11B	110.2
C3—C2—C1	119.4 (3)	H11A—C11—H11B	108.5
C4—C3—C2	120.0	N1—C12—C13	112.3 (3)
C4—C3—H3	120.0	N1—C12—C9	109.9 (3)
C2—C3—H3	120.0	C13—C12—C9	111.9 (3)
C3—C4—C5	120.0	N1—C12—H12	107.5
C3—C4—H4	120.0	C13—C12—H12	107.5
C5—C4—H4	120.0	C9—C12—H12	107.5
C14—C13—C18	120.0	C29—O2—C26	107.6 (3)
C14—C13—C12	118.6 (2)	C23—N2—C30	117.3 (3)
C18—C13—C12	121.3 (2)	C23—N2—H2	121.3
C15—C14—C13	120.0	C30—N2—H2	121.3
C15—C14—H14	120.0	C20—C19—H19A	109.5
C13—C14—H14	120.0	C20—C19—H19B	109.5
C14—C15—C16	120.0	H19A—C19—H19B	109.5
C14—C15—H15	120.0	C20—C19—H19C	109.5
C16—C15—H15	120.0	H19A—C19—H19C	109.5
C17—C16—C15	120.0	H19B—C19—H19C	109.5
C17—C16—H16	120.0	O2—C26—C24	110.7 (3)
C15—C16—H16	120.0	O2—C26—C27	104.3 (3)
C16—C17—C18	120.0	C24—C26—C27	116.0 (3)
C16—C17—H17	120.0	O2—C26—H26	108.6
C18—C17—H17	120.0	C24—C26—H26	108.6
C17—C18—C13	120.0	C27—C26—H26	108.6
C17—C18—H18	120.0	C30—C27—C26	109.1 (3)
C13—C18—H18	120.0	C30—C27—C28	114.1 (4)
C36—C31—C32	120.0	C26—C27—C28	102.9 (3)
C36—C31—C30	120.5 (3)	C30—C27—H27	110.2
C32—C31—C30	119.5 (3)	C26—C27—H27	110.2
C31—C36—C35	120.0	C28—C27—H27	110.2
C31—C36—H36	120.0	C29—C28—C27	104.9 (3)
C35—C36—H36	120.0	C29—C28—H28A	110.8
C34—C35—C36	120.0	C27—C28—H28A	110.8
C34—C35—H35	120.0	C29—C28—H28B	110.8
C36—C35—H35	120.0	C27—C28—H28B	110.8
C35—C34—C33	120.0	H28A—C28—H28B	108.8
C35—C34—H34	120.0	O2—C29—C28	108.5 (4)
C33—C34—H34	120.0	O2—C29—H29A	110.0
C32—C33—C34	120.0	C28—C29—H29A	110.0
C32—C33—H33	120.0	O2—C29—H29B	110.0
C34—C33—H33	120.0	C28—C29—H29B	110.0
C33—C32—C31	120.0	H29A—C29—H29B	108.4
C33—C32—H32	120.0	N2—C30—C31	110.4 (3)
C31—C32—H32	120.0	N2—C30—C27	108.0 (3)
C8—O1—C11	110.5 (3)	C31—C30—C27	116.2 (3)
C5—N1—C12	119.9 (3)	N2—C30—H30	107.3
C5—N1—H1	120.1	C31—C30—H30	107.3
C12—N1—H1	120.1	C27—C30—H30	107.3
			
N2—C23—C24—C25	177.6 (2)	C7—C6—C8—O1	53.0 (4)
C22—C23—C24—C25	0.0	C5—C6—C8—C9	−15.4 (4)
N2—C23—C24—C26	−5.5 (3)	C7—C6—C8—C9	168.8 (3)
C22—C23—C24—C26	176.8 (3)	O1—C8—C9—C10	32.1 (4)
C23—C24—C25—C20	0.0	C6—C8—C9—C10	−86.8 (4)
C26—C24—C25—C20	−176.9 (3)	O1—C8—C9—C12	157.0 (3)
C24—C25—C20—C21	0.0	C6—C8—C9—C12	38.1 (4)
C24—C25—C20—C19	−177.9 (3)	C8—C9—C10—C11	−28.3 (4)
C25—C20—C21—C22	0.0	C12—C9—C10—C11	−152.3 (4)
C19—C20—C21—C22	177.9 (3)	C8—O1—C11—C10	5.3 (5)
C20—C21—C22—C23	0.0	C9—C10—C11—O1	15.4 (5)
N2—C23—C22—C21	−177.6 (2)	C5—N1—C12—C13	171.8 (3)
C24—C23—C22—C21	0.0	C5—N1—C12—C9	46.5 (4)
C4—C5—C6—C7	0.0	C14—C13—C12—N1	148.6 (2)
N1—C5—C6—C7	−176.7 (3)	C18—C13—C12—N1	−34.7 (4)
C4—C5—C6—C8	−175.7 (3)	C14—C13—C12—C9	−87.2 (3)
N1—C5—C6—C8	7.6 (3)	C18—C13—C12—C9	89.4 (3)
C5—C6—C7—C2	0.0	C10—C9—C12—N1	64.8 (4)
C8—C6—C7—C2	175.8 (3)	C8—C9—C12—N1	−53.2 (4)
C6—C7—C2—C3	0.0	C10—C9—C12—C13	−60.6 (4)
C6—C7—C2—C1	−179.9 (3)	C8—C9—C12—C13	−178.7 (3)
C7—C2—C3—C4	0.0	C24—C23—N2—C30	−25.0 (4)
C1—C2—C3—C4	179.9 (3)	C22—C23—N2—C30	152.6 (2)
C2—C3—C4—C5	0.0	C29—O2—C26—C24	−160.0 (3)
C6—C5—C4—C3	0.0	C29—O2—C26—C27	−34.6 (4)
N1—C5—C4—C3	176.8 (3)	C25—C24—C26—O2	−64.7 (3)
C18—C13—C14—C15	0.0	C23—C24—C26—O2	118.5 (3)
C12—C13—C14—C15	176.7 (3)	C25—C24—C26—C27	176.9 (3)
C13—C14—C15—C16	0.0	C23—C24—C26—C27	0.1 (4)
C14—C15—C16—C17	0.0	O2—C26—C27—C30	−89.9 (4)
C15—C16—C17—C18	0.0	C24—C26—C27—C30	32.0 (5)
C16—C17—C18—C13	0.0	O2—C26—C27—C28	31.6 (4)
C14—C13—C18—C17	0.0	C24—C26—C27—C28	153.5 (4)
C12—C13—C18—C17	−176.6 (3)	C30—C27—C28—C29	100.3 (4)
C32—C31—C36—C35	0.0	C26—C27—C28—C29	−17.7 (5)
C30—C31—C36—C35	177.7 (3)	C26—O2—C29—C28	23.5 (5)
C31—C36—C35—C34	0.0	C27—C28—C29—O2	−2.5 (6)
C36—C35—C34—C33	0.0	C23—N2—C30—C31	−174.1 (3)
C35—C34—C33—C32	0.0	C23—N2—C30—C27	57.8 (4)
C34—C33—C32—C31	0.0	C36—C31—C30—N2	−64.4 (3)
C36—C31—C32—C33	0.0	C32—C31—C30—N2	113.3 (3)
C30—C31—C32—C33	−177.7 (3)	C36—C31—C30—C27	59.0 (4)
C6—C5—N1—C12	−24.4 (4)	C32—C31—C30—C27	−123.2 (3)
C4—C5—N1—C12	158.8 (3)	C26—C27—C30—N2	−58.8 (4)
C11—O1—C8—C9	−23.5 (5)	C28—C27—C30—N2	−173.2 (3)
C11—O1—C8—C6	96.6 (4)	C26—C27—C30—C31	176.5 (3)
C5—C6—C8—O1	−131.3 (3)	C28—C27—C30—C31	62.1 (4)

### Hydrogen-bond geometry (Å, °)

**Table:**

*D*—H···*A*	*D*—H	H···*A*	*D*···*A*	*D*—H···*A*
N1—H1···O2	0.86	2.41	2.959 (4)	122
N2—H2···O1^i^	0.86	2.15	2.934 (4)	151

Symmetry codes: (i) −*x*+1, *y*−1/2, −*z*+1/2.
